# The glutaredoxin mono- and di-thiol mechanisms for deglutathionylation are functionally equivalent: implications for redox systems biology

**DOI:** 10.1042/BSR20140157

**Published:** 2015-02-25

**Authors:** Lefentse N. Mashamaite, Johann M. Rohwer, Ché S. Pillay

**Affiliations:** *School of Life Sciences, University of KwaZulu-Natal, Carbis Road, Pietermaritzburg, 3201, South Africa; †Department of Biochemistry, Stellenbosch University, Private Bag X1, Matieland, 7602 Stellenbosch, South Africa

**Keywords:** kinetics, redox regulation, redoxin, thiol, Grx, glutaredoxin, GrxSS, oxidized glutaredoxin, GrxSSGSH, glutaredoxin–GSH mixed disulphide, GSSG, oxidized glutathione, HED, β-hydroxyethyl disulphide, PySCeS, Python Simulator of Cellular Systems, ROS, reactive oxygen species

## Abstract

Glutathionylation plays a central role in cellular redox regulation and anti-oxidative defence. Grx (Glutaredoxins) are primarily responsible for reversing glutathionylation and their activity therefore affects a range of cellular processes, making them prime candidates for computational systems biology studies. However, two distinct kinetic mechanisms involving either one (monothiol) or both (dithiol) active-site cysteines have been proposed for their deglutathionylation activity and initial studies predicted that computational models based on either of these mechanisms will have different structural and kinetic properties. Further, a number of other discrepancies including the relative activity of active-site mutants and contrasting reciprocal plot kinetics have also been reported for these redoxins. Using kinetic modelling, we show that the dithiol and monothiol mechanisms are identical and, we were also able to explain much of the discrepant data found within the literature on Grx activity and kinetics. Moreover, our results have revealed how an apparently futile side-reaction in the monothiol mechanism may play a significant role in regulating Grx activity *in vivo*.

## INTRODUCTION

The Grx (glutaredoxin) system was initially described as an alternate electron donor to ribonucleotide reductase in *Escherichia coli* mutants lacking thioredoxin [1]. In this system, reducing equivalents from NADPH were transferred to the abundant cellular thiol glutathione (GSH) by glutathione reductase. GSH in turn reduced a glutaredoxin, Grx1, which then reduced ribonucleotide reductase [2–4]. This coupled series of redox reactions were characterized by the transfer of two reducing equivalents per reaction and subsequently this Grx and the Grxs in other species, were shown to use this dithiol mechanism to reduce protein targets involved in several metabolic and regulatory processes [2,3,5].

Grxs were also found to be capable of reducing the mixed disulphides formed between GSH and protein thiols. These disulphides can be generated by several mechanisms, including the ROS (reactive oxygen species)-dependent activation of protein and GSH thiol groups [6], and glutathionylated proteins are therefore considered biomarkers of oxidative stress [7]. Under these conditions, glutathionylation protects labile protein thiols from hyper-oxidation but can also affect the structure and activity of target proteins [7–9]. Grxs, together with thioredoxins in some species [10], are primarily responsible for reversing this process [2,3]. However, even under normoxic conditions the glutathionylation/deglutathionylation cycle appears to be an important post-translational redox regulatory mechanism affecting a number of critical cellular processes [6–8].

**Figure 1:**
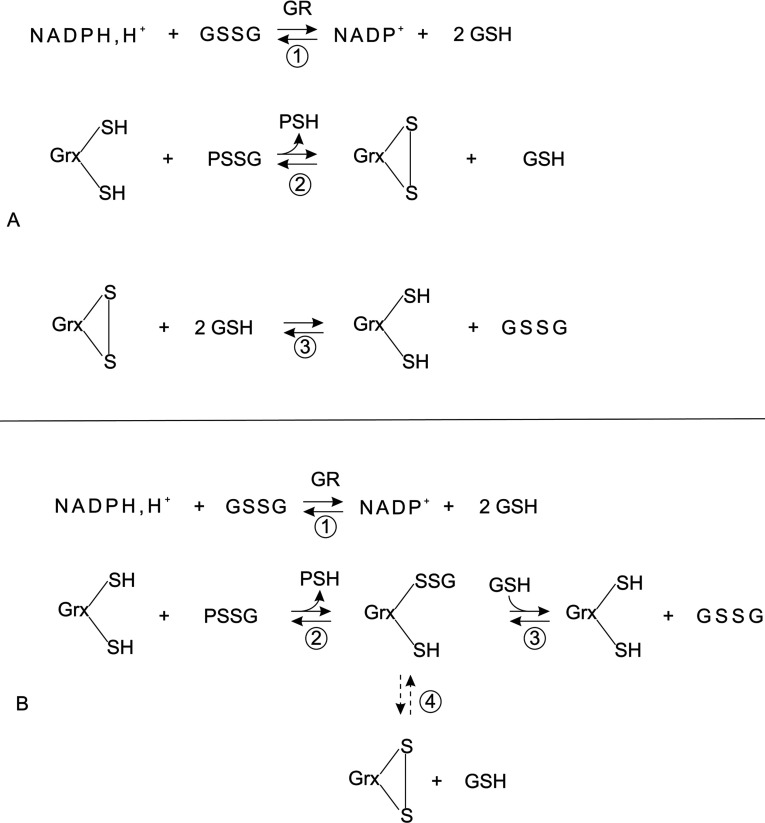
A comparison of the Grx dithiol and monothiol mechanisms for deglutathionylation In the dithiol mechanism (**A**), the N-terminal active-site cysteine of glutaredoxin (Grx(SH)_2_) initiates a nucleophilic attack on the glutathionylated protein substrate (PSSG) resulting in a mixed disulphide that is attacked by the second C-terminal active-site cysteine releasing the reduced protein (protein SH) and GrxSS (reaction 2) which is subsequently reduced by two GSH molecules (reaction 3). In the monothiol mechanism (**B**), the N-terminal thiolate anion forms a GrxSSGSH releasing the reduced protein (reaction 2). This mixed disulphide is reduced by glutathione, regenerating active Grx (reaction 3). A side-reaction resulting in the formation of GrxSS detracts from catalysis. (reaction 4). GSSG is regenerated by glutathione reductase in both mechanisms (reaction 1).

Given the importance of deglutathionylation in regulating these processes, computational systems biology approaches could provide insights into the key regulatory features of this network of reactions [11]. However, two contrasting mechanisms have been proposed for the deglutathionylation activity of Grxs (Figure 1) [3,12–14]. The dithiol mechanism resembles the disulphide reduction mechanism with both active-site cysteine residues used to reduce glutathionylated substrates, resulting in the formation of GrxSS (oxidized glutaredoxin) which is reduced by two GSH molecules (Figure 1A) [3,12–14]. In the monothiol mechanism on the other hand, only the N-terminal cysteine and a single glutathione molecule are required for each reduction event and the formation of GrxSS is considered a side-reaction that detracts from catalysis (Figure 1B) [3,12–14].

Although the monothiol mechanism is generally considered to be the deglutathionylation mechanism used by Grxs [3,12–14], the data supporting both these mechanisms have sometimes been contradictory and enigmatic. For example, as GrxSS represents a dead-end species in the monothiol mechanism, a mutation of the C-terminal active-site cysteine to a serine (CXXC→S) should increase the specific deglutathionylation rate [12,15]. This was indeed the case for some Grxs (see, for example, [16]) but for others, this mutation decreased the deglutathionylation rate considerably (see, for example, [17,18]). Other studies apparently support the dithiol mechanism as the deglutathionylation rate has shown a sigmoidal dependence on glutathione [17] and, GrxSS is a prominent species in quenched-flow trapping experiments [17] and has been detected *in vivo* [19]. Further and somewhat surprisingly, most native monothiol Grxs cannot catalyse deglutathionylation reactions [3,13]. In a previous study, we developed a computational model of the *E. coli* Grx system based on the dithiol mechanism. This model fitted an *in vitro* kinetic dataset and was able to successfully predict two independent kinetic datasets, confirming its accuracy [20]. However, given the uncertainty on the deglutathionylation mechanism used by Grx, it was not clear whether this modelling approach could be extended to other Grxs.

The double reciprocal plot patterns obtained with Grxs have also proved to be a source of confusion with some plots showing that deglutathionylation follows a ping-pong kinetic mechanism while other studies have reported a sequential kinetic mechanism with the model substrate HED (β-hydroxyethyl disulphide) [21–23]. In addition, secondary plot data have indicated that some Grxs may have infinite Michaelis–Menten binding constants [16] implying that mass action kinetics could be used to describe the Grx redox cycle [20]. Collectively, these results have made modelling Grxs for computational systems biology extremely difficult as it not clear whether to model deglutathionylation with a mono- or dithiol mechanism especially, as a comparative analysis of these mechanisms has revealed that computational models based on either of these mechanisms would have distinct properties [11]. Further, given the contradictory findings reported for Grx kinetics, it is also not certain whether these models would be accurate. In the present study, we used a number of modelling techniques to resolve both the question of which the deglutathionylation mechanism should be used in computational models, and clarify some of the contradictory data on Grx kinetics which have been presented in the literature.

## MATERIALS AND METHODS

### Kinetic modelling

Kinetic modelling experiments were carried out using the PySCeS (Python Simulator for Cellular Systems) [24] as described previously [20,25]. In certain modelling experiments, we used a validated *E. coli* Grx model from a previous study [20] which was based on *in vitro* kinetic data obtained from Peltoniemi et al. [17]. For the kinetic fitting experiments described in this study, non-linear least squares regression with the Levenberg–Marquardt algorithm from SciPy (http://www.scipy.org) was used to fit data to models of the yeast Grx1 and Grx2 systems [26]. Note, in this approach the entire Grx system of reactions was initialized in PySCeS and then fitted to the *in vitro* kinetic datasets [20]. Kinetic parameters for the models were obtained from the literature or from the BRENDA database (available at http://www.brenda-enzymes.org) [27]. The kinetic parameters and rate expressions used in the fitting experiments together with PySCeS and SBML formatted models [28] are available in Table 1 or in the Supplementary information.

**Table 1:** Kinetic parameters and species concentrations used for the yeast Grx 1 and Grx 2 models of Li *et al.* [26].

	Value		
	Grx1	Grx2	Reference
**Metabolite**			
NADPH	250 μM	250 μM	[26]
NADP	1 μM	1 μM	[26]
GSH	998 μM	998 μM	[26]
GSSG	1 μM	1 μM	[26]
HED	70 μM	70 μM	[26]
**Redoxin**			
Grx(SH)_2_	0.12 μM	0.02 μM	[26]
Grx(SS)	0.12 μM	0.02 μM	[26]
**Glutathione reductase**			
*K*_NADPH_	15 μM	15 μM	[34]
*K*_GSSG_	74.6 μM	74.6 μM	[34]
*k*_cat_	900 s^−1^	900 s^−1^	[34]
[Glutathione reductase]	0.02 μM	0.02 μM	[26]

## RESULTS

### The mono- and di- thiol mechanisms for deglutathionylation are equivalent

**Figure 2:**
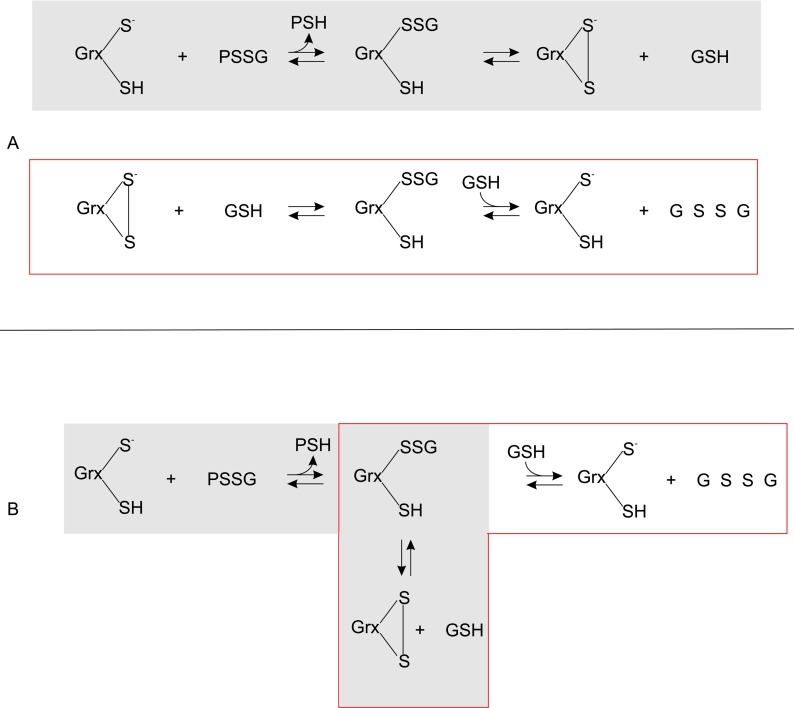
The Grx dithiol and monothiol mechanisms for deglutathionylation are identical Once the the glutaredoxin-GSH mixed disulfide (GrxSSGSH) intermediate is included in the dithiol mechanism (**A**) and oxidized glutaredoxin (GrxSS) formation is considered part of the monothiol mechanism (**B**), then the reaction schemes for these mechanisms are identical.

**Figure 3:**
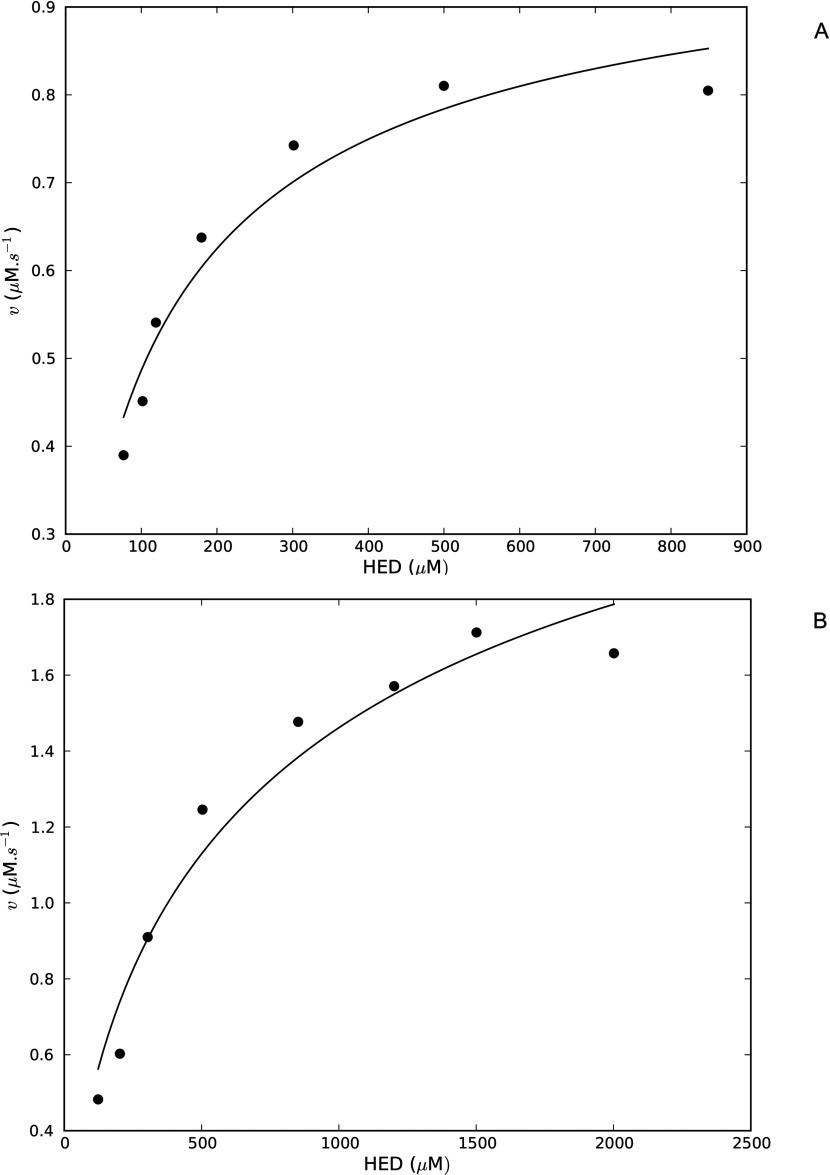
Kinetic models based on the Grx dithiol mechanism can successfully describe *in vitro* datasets Yeast Grx dithiol models were fitted to datasets describing the reduction of HED by Grx 1 (**A**) and Grx 2 (**B**) [26]. For the Grx 1 dataset (**A**), the fitted rate constants for glutaredoxin and HED reduction were 4.23±0.30×10^−6^ μM^−2^·s^−1^ and 0.073±0.011 μM^−1^·s^−1^ respectively, and the goodness of fit was assessed by an *r*^2^ value of 0.94. Rate constants for glutaredoxin and HED reduction of 6.74±0.96×10^−5^ μM^−2^·s^−1^ and 0.252±0.045 μM^−1^·s^−1^ respectively, were obtained for the Grx2 dataset with an *r*^2^ value of 0.97.

We sought to answer a central question on the modelling of Grxs for systems biology studies: which mechanism should be used in computational models of the Grx system? Kinetic models based on the mono- and dithiol mechanisms are expected to have distinct stoichiometric matrices as the GrxSSGSH (mixed-disulphide glutaredoxin–GSH) species is apparently found in the monothiol, but not the dithiol mechanism (Figure 1) [11]. However, if the intermediate steps leading to the reduction of the glutathionylated substrate (PSSG) and the subsequent reduction of GrxSS are considered in the dithiol mechanism (reactions 2–3, Figure 1A), then the GrxSSGSH species is featured in both mechanisms (Figure 2A). In fact, once the side-reaction involving GrxSS is also included in the monothiol mechanism (Figure 2B), it is clear that the mono- and dithiol mechanisms are identical (Figure 2). Thus, as we previously proposed with our *E. coli* glutaredoxin model [20], deglutathionylation can be accurately modelled with the dithiol mechanism (Figure 1A) in computational systems biology models. In this approach, the GrxSSGSH species was not explicitly modelled as quenched-flow experiments that showed that this species rapidly formed GrxSS and was therefore not recovered in these experiments (see, for example, [17]). To extend our previous fitting results [20], two *in vitro* kinetic datasets were fitted to models of the yeast Grx system (Table 1, [26]) and in both cases, good fits of the data were obtained *(r*^2^≥0.94, Figure 3). However, our models showed a poorer fit of the data at the high and low concentration range of HED used in this assay. This deviation was expected as the HED reaction as reversible (see below) and irreversible mass action kinetics were used in our models because the equilibrium constant for this reaction was not known.

### The differences in activity between wild-type and mutant Grxs depends on their rate constants for GSH oxidation and on the GSH concentration

We next aimed to determine why the activity of active-site Grx mutants appeared to be higher than the wild-type redoxins in some cases [16] but not in others [17,18]. To aid this analysis we used core mathematical modelling to compare wild-type and mutant redoxins. In these modelling experiments, we focused on the Grx redox cycle as the glutathione reductase reaction was common to both systems and we used the dithiol mechanism to represent the wild-type Grx (Scheme I).

Scheme I: wild-type Grx

(1) GrxSH2+PSSGK1⟶GrxSS+GSH

(2) GrSS+2GSHk2⟶GrxSH2+GSSG

In this analysis, these reactions were described with irreversible mass-action kinetics [20]. Eqns (1) and (2) can therefore be described by a series of rate equations and the Grx moiety couple can be related to its sum [eqns (3)–(5)]:

(3) $$v_{1}=k_{1}.\mathrm{PSSG}.\mathrm{Grx}{\left(\mathrm{SH}\right)}_{2}$$

(4) $$v_{2}=k_{2}.\mathrm{GrxSS}.{\mathrm{GSH}}^{2}$$

(5) $$\mathrm{Grx}{\left(\mathrm{SH}\right)}_{2}+\mathrm{GrxSS}={\mathrm{Grx}}_{\mathrm{tot}}$$

At steady state *v*_1_=*v*_2_ and therefore:

(6) $$k_{1}.\mathrm{PSSG}.\mathrm{Grx}{\left(\mathrm{SH}\right)}_{2}=k_{2}.\mathrm{GrxSS}{.\mathrm{GSH}}^{2}$$

Eqn (6) can be rearranged to yield:

(7) $$\mathrm{GrxSS}=\frac{k_{1}.\mathrm{PSSG}{.\mathrm{Grx}}_{\mathrm{tot}}}{k_{1}.\mathrm{PSSG}+k_{2}.{\mathrm{GSH}}^{2}}$$

This equation can then be substituted into eqn (4) to give the following expression for the rate of the wild-type (wt) mechanism:

(8) $$v_{2\mathrm{wt}}=\frac{\mathrm{PSSG}{.\mathrm{Grx}}_{\mathrm{tot}}{.\mathrm{GSH}}^{2}}{\frac{\mathrm{PSSG}}{k_{2}}+\frac{{\mathrm{GSH}}^{2}}{k_{1}}}$$

Eqn (8) can be further simplified to yield:

(9) $$v_{2\mathrm{wt}}=\frac{{\mathrm{Grx}}_{\mathrm{tot}}}{\frac{1}{k_{2}.{\mathrm{GSH}}^{2}}+\frac{1}{k_{1}.\mathrm{PSSG}}}$$

A similar reaction scheme and set of equations can be derived for the mutant redoxin:

Scheme II: mutant Grx

(10) GrxSH+PSSGk1⟶GrxSSG+PSH

(11) GrxSSG+GSHk2′⟶GrxSH+GSSG

Scheme II can be described by the following equations:

(12) $$v_{1}=k_{1}.\mathrm{PSSG}.\mathrm{GrxSH}$$

(13) $$v_{2}=k_{2}^{\prime }.\mathrm{GrxSSG}.\mathrm{GSH}$$

(14) $$\mathrm{GrsxSSG}+\mathrm{GrxSH}={\mathrm{Grx}}_{\mathrm{tot}}$$

Note that the rate constants for the GSH oxidation (*v*_2_) are different for the wild-type (*k*_2_, M^−2^·min^−1^) and mutant (*k*_2_′, M^−1^·min^−1^) redoxins and are therefore not directly comparable. Further while the Grx mutant uses a single active-site cysteine, its mechanism is distinct from the monothiol mechanism as described above which allows the formation of GrxSS (Figure 2). As for the wild-type mechanism [eqn (9)], it can be shown that the rate expression for the mutant (mu) redoxin can be described by the following equation:

(15) $$v_{2\mathrm{mu}}=\frac{{\mathrm{Grx}}_{\mathrm{tot}}}{\frac{1}{k_{2}^{\prime }.\mathrm{GSH}}+\frac{1}{k_{1}.\mathrm{PSSG}}}$$

To compare the wild-type and mutant mechanisms, eqn (9) can be divided by eqn (15) to yield a ratio of their rates:

(16) $$\frac{v_{2\mathrm{wt}}}{v_{2\mathrm{mu}}}=\frac{\frac{1}{k_{2}^{\prime }.\mathrm{GSH}}+\frac{1}{k_{1}.\mathrm{PSSG}}}{\frac{1}{k_{2}.{\mathrm{GSH}}^{2}}+\frac{1}{k_{1}.\mathrm{PSSG}}}$$

The term *k*_1_*.PSSG* represents the reduction of PSSG and at high substrate concentrations (*k*_1_.*PSSG*>>1), eqn (16) simplifies to:

(17) $$\frac{v_{2\mathrm{wt}}}{v_{2\mathrm{mu}}}=\frac{k_{2}.\mathrm{GSH}}{k_{2}^{\prime }}$$

This result offered a number of insights into the rates of wild-type and mutant Grxs. First, any differences in the rates between the wild-type and mutant Grxs at high substrate concentrations can be explained solely by differences in the rate constants for GSH oxidation (*k*_2_.*GSH* vs. *k*_2_') in the two reaction schema. The results from *in vitro* studies have shown that this step is indeed the rate-limiting step during deglutathionylation [29]. Secondly, if a mutation of the Grx C-terminal active-site cysteine resulted in an increased rate of glutathione oxidation for a particular GSH concentration (*k*_2_′>*k*_2_.*GSH*), then that mutant redoxin could have a higher activity than its corresponding wild-type redoxin. However, with increases in the glutathione concentration, eqn (17) predicts that that the relative rate of wild-type to mutant will increase. This has been confirmed by *in vitro* data with human Grx2 where mutant and wild-type rates became equivalent at high glutathione concentrations (cf. Figure 3B, [16]).

### Computational models reveal the basis behind the anomalous double-reciprocal plot patterns obtained for Grxs

Kinetic data on Grxs have shown discrepant behaviour with some studies reporting reciprocal plot data with GSH yielded non-linear plots (see, for example, [17]) while other studies have reported linear plots (see, for example, [16,23]). Further, some studies have shown that deglutathionylation reactions apparently follow a sequential mechanism (see, for example, [21–23]), while other studies have reported a ping-pong mechanism for this reaction (see, for example, [16]). To understand these behaviours, we rearranged the simplified rate expression for the Grx system [eqn (18)] and compared it with the standard ping-pong rate expression [eqn (19)]

(18) $$\frac{1}{v_{2\mathrm{wt}}}=\frac{1}{{\mathrm{Grx}}_{\mathrm{tot}}.k_{1}}\left(\frac{1}{\mathrm{PSSG}}\right)+\frac{1}{{\mathrm{Grx}}_{\mathrm{tot}}.k_{2}}\left(\frac{1}{{\mathrm{GSH}}^{2}}\right)$$

(19) $$\frac{1}{v}=\frac{k_{a}}{V}\left(\frac{1}{a}\right)+\frac{1}{V}\left(\frac{k_{b}}{b}+1\right)$$

This analysis showed that plotting the reciprocal rate against the reciprocal of the glutathionylated substrate concentration (*PSSG*) at different glutathione concentrations would result in parallel lines with a gradient of 1/Grx_tot_.*k*_1_ [eqn (18)]. On the other hand, eqn (18) also predicts a quadratic relationship between 1/*v*_2wt_ and 1/*GSH*, and plotting the reciprocal rate against the reciprocal GSH concentration would result in a non-linear response, especially at high concentrations of GSH (low values of 1/*GSH*).

To substantiate this result we used our previously described *E. coli* Grx kinetic model [20] to develop reciprocal plots of rate against glutathionylated peptide substrate (*PSSG*) and GSH. The kinetic model consisted of three reactions (Scheme III) corresponding to the dithiol mechanism (Figure 1A). In this model, glutathione reductase [*GR*, eqn (20)] was modelled with an irreversible, two-substrate generic rate expression [30], whereas GSH-oxidation and the reduction of the deglutathionylated substrate were modelled with mass-action kinetics [eqns (20)–(22)] as described previously [20].

Scheme III: *E. coli* Grx kinetic model

(20) NADPH+GSSGGR⟶2GSH+NADP

(21) GrxSS+2GSHk2⟶GrxSH2+GSSG

(22) GrxSH2+PSSGk3⟶GrxSS+PSH+GSH

**Figure 4:**
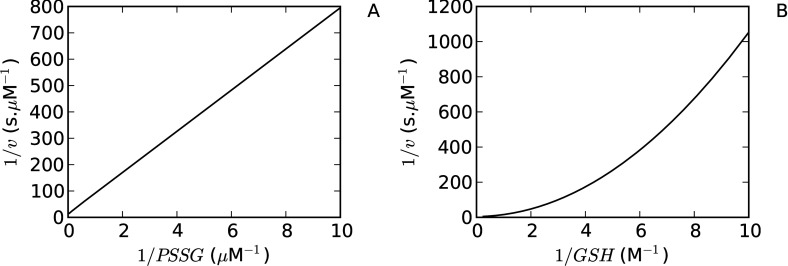
The contrasting reciprocal plot kinetics for Grx substrates can be explained using a Grx dithiol computational model Analysis of a validated *E. coli* glutaredoxin model [20] revealed that double-reciprocal plots for a glutathionylated substrate (PSSG) (**A**) or GSH (**B**) were expected to show distinct responses to changes in substrate concentration. In (**A**) the PSSG concentration was varied from 0.1–20 μM with a constant GSH concentration (1.0 mM) while in (**B**) the GSH concentration was varied from 0.1–4.0 mM with a constant PSSG concentration (5 μM).

As expected, a linear response was obtained with the glutathionylated substrate (Figure 4A), while a non-linear response was obtained with GSH in reciprocal plots (Figure 4B) which was consistent with the previously described *in vitro* data (cf. Figure 3, [17]). In studies where a linear response to GSH have been reported (see, for example, [16,23]), the GSH concentrations used were generally within the quasi-linear region of the double-reciprocal plot (Figure 4B) which resulted in an apparent linear response to 1/*GSH*.

Interestingly, in substrate saturation experiments, wild-type Grxs have shown a sigmoidal response to GSH, while mutant Grxs have shown a hyperbolic response to this thiol (cf. [17]). To precisely describe this effect using our mathematical model, eqn (8) was rearranged to highlight the rate dependence on GSH:

(23) $$\begin{array}{ccc} v_{\mathrm{wt}} & = & \frac{{\mathrm{Grx}}_{\mathrm{tot}}.k_{1}.k_{2}.\mathrm{PSSG}{.\mathrm{GSH}}^{2}}{k_{1}.\mathrm{PSSG}+k_{2}.{\mathrm{GSH}}^{2}}\\ & = & \frac{{\mathrm{Grx}}_{\mathrm{tot}}.k_{1}.\mathrm{PSSG}\frac{{\mathrm{GSH}}^{2}}{k_{1}.\mathrm{PSSG}/k_{2}}}{1+\frac{{\mathrm{GSH}}^{2}}{k_{1}.\mathrm{PSSG}/k_{2}}}\\ & = & \frac{V.\sigma^{2}}{1+\sigma^{2}} \end{array}$$

with *V=Grx*_tot_*.k*_1_*.PSSG*, σ=*GSH/GSH*_0.5_ and *GSH*_0.5_ =(*k*_1_.*PSSG/k*_2_)^0.5^. Thus, eqn (23) was a form of the Hill equation with a maximal rate (*V*), a half-saturation constant (*GSH*_0.5_) and a Hill-coefficient of two. Similarly, it can be shown that the dependence on GSH for mutant Grxs could be described with:

(24) $$v_{\mathrm{mu}}=\frac{V.\sigma }{1+\sigma }$$

where *V* and σ are defined as above but *GSH*_0.5_=*k*_1_.*PSSG/k*_2_^′^. A comparison of eqns (23) and (24) showed that a quadratic relationship would be expected between rate and GSH concentration for the wild-type Grx [eqn (23)] but a hyperbolic relationship with glutathione would be expected for the mutant Grx [eqn (24)] which is in agreement with *in vitro* data (cf. Figures 3C and 4C [17]). Further, a model of the Grx system which was fitted to an independent dataset also showed this non-linear response to GSH concentrations [20].

**Figure 5:**
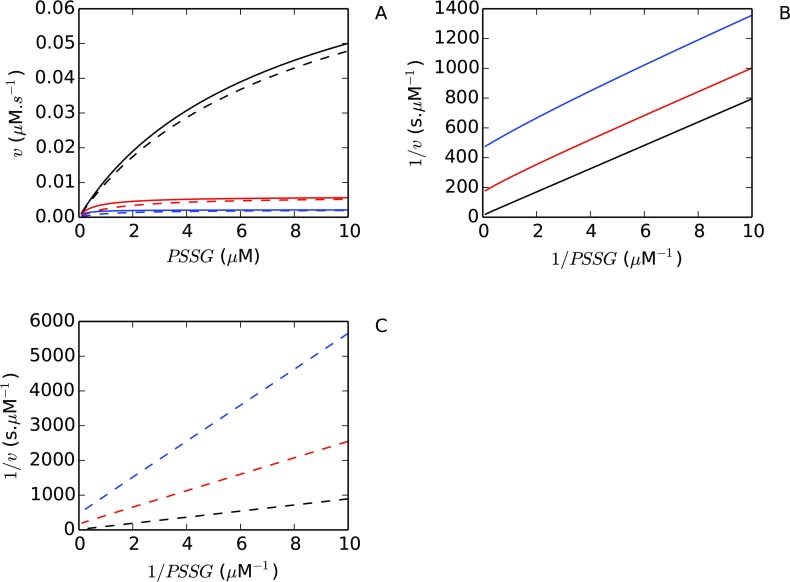
Double reciprocal plots of the Grx system can show ping-pong or sequential kinetic patterns depending on the reversibility of the deglutathionylation reaction An *E. coli* Grx computational model [20] with the deglutathionylation of a substrate (PSSG) modelled with irreversible (solid) or reversible mass action kinetics (dashes) at varying GSH concentrations of 150 (blue), 250 (red) and 1000 (black) μM was analysed (**A**). Reciprocal plots of the models revealed a ping-pong kinetic pattern (**B**) when deglutathionylation was modelled with irreversible kinetics but a sequential pattern was obtained when this reaction was modelled with reversible kinetics (**C**).

We next considered the reasons behind the sequential and ping-pong kinetic patterns obtained with Grxs especially as eqn (18) predicted that parallel and not convergent lines should be expected with glutathionylated substrates. It had been noted that the sequential kinetic pattern in Grx reciprocal plots were obtained with the substrate HED [21–23]. In this assay, HED is spontaneously reduced by glutathione to form a mixed disulphide that is then reduced by Grx to yield β-mercaptoethanol and GSSG (oxidized glutathione). As these products can react with each other to reform the substrate [31], we wondered how the reciprocal plot data would be affected if the reduction of the glutathionylated substrate was modelled with reversible kinetics. To test this hypothesis, we modified our *E. coli* Grx kinetic model by making just the deglutathionylation reaction [eqn (22), Scheme III] reversible, with an equilibrium constant equal to one micromolar. Simulation of both models (Figure 5A) revealed that when this reaction was made reversible, the reciprocal kinetic pattern obtained in the original model altered from an apparent ping-pong mechanism with parallel lines (Figure 5B) to an apparent sequential mechanism with converging lines (Figure 5C). At low concentrations of glutathionylated substrate there was a marked difference in the rates obtained with different concentrations of glutathione in the reversible deglutathionylation model (dashed lines, Figure 5A). These differences became exaggerated in the reciprocal plot, leading to the apparently converging kinetic pattern observed in this plot (Figure 5C).

## DISCUSSION

It has been established that the Grxs play a central role in the redox regulation of several metabolic, transcriptional and structural cellular processes under a range of normoxic, hypoxic and hyperoxic conditions (reviewed in [14]). However, uncertainty over the monothiol and dithiol catalytic mechanisms used by Grxs (Figure 1) and conflicting descriptions of Grx activity and kinetics have limited our understanding of Grx activity in these processes and alternate kinetic models for Grx activity have been postulated by other groups (see, for example, [3,32]). For computational systems biology studies in particular, these contradictory descriptions had a critical limitation as models built with the monothiol or dithiol mechanism would be expected to give different results with the same set of input parameters [11].

Our results revealed that the dithiol and monothiol mechanisms are in fact identical (Figure 2) and this result, together with kinetic fitting results from a previous study [20] and from this study (Figure 3), showed that Grxs can be accurately modelled with a dithiol mechanism in computational systems biology studies. Further mathematical analyses provided the rationale behind the confusing data presented on the activity of Grx active-site mutants. Our results showed that the relative rates of wild-type and mutant Grxs depended critically on the rate constant for Grx-dependent GSH oxidation within these systems [eqn (17)] and on the GSH concentration in the assay. An interesting prediction made by our analysis was that the relative rate of a wild-type redoxin (compared with the mutant) was dependent on the GSH concentration and with increasing concentrations of GSH, this relative rate would increase which has been confirmed *in vitro* [16].

We were also able to provide an explanation for the sequential and ping-pong kinetic patterns which have been obtained in double reciprocal plots with Grxs. Our mathematical modelling results show that a ping-pong kinetic pattern would be expected for most glutathionylated substrates [eqn (18)]. However, if the deglutathionylation reaction was significantly reversible, our results showed that a sequential pattern would be obtained in double reciprocal plots (Figure 5). Finally, our results have also revealed the conditions under which reciprocal plot data with GSH would result in linear and non-linear plots (Figure 4). Thus, by focusing on the dynamics of the Grx *system*, many of the discrepant results reported in the biochemical literature on Grx activity could be explained.

**Figure 6:**
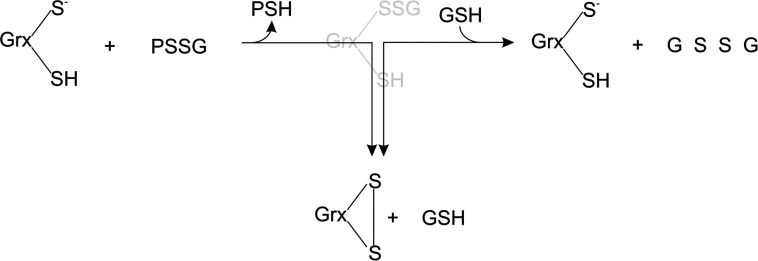
The formation of GrxSS may be a mechanism to prevent deglutathionylation in the presence of ROS See the text for details.

Although this analysis has answered several questions on Grx kinetics, it has also raised an intriguing observation. It appears that the reduction of glutathionylated substrates proceeds via an apparently futile side-reaction involving GrxSS (Figure 6). This observation was also described by Peltoniemi et al. [17] who speculated that the formation of GrxSS *in vivo* may be a mechanism to prevent Grx hyper-oxidation during oxidative stress. Once the oxidative stress was relieved, there would be a corresponding increase in cellular GSH levels and active Grx would then be available [17]. We suggest that this mechanism may serve as an additional function. A critical role for deglutathionylation is to protect labile cysteine residues from ROS-induced oxidation and therefore deglutathionylation under these conditions may actually expose these thiols to oxidative damage. However, the formation of GrxSS in the presence of relatively high GSSG and low GSH concentrations [17] provisionally inactivates the redoxin until its activity is required.

This mechanism has two potentially relevant implications for cellular redox regulation. First, the formation and persistence of oxidized cytoplasmic Grx *in vivo* may represent an important cellular redox sensor and therefore clinical biomarker for oxidative stress. This biomarker, while more difficult to assay, has an advantage over GSH/GSSG measurements, in that it may be less affected by compartment mixing during cell lysis [33]. Secondly, this result and other results on ultrasensitivity in the thioredoxin system [25] have emphasized how the dynamics of redoxin systems allow for regulatory behaviours that cannot be predicted by considering the system components in isolation. It would be expected that computational modelling will play a key role in elucidating further dynamic behaviour within these systems and this work represents an important step in realizing this goal.

## Online data

Supplementary data
