# Supplementary material for: The glutaredoxin mono- and di-thiol mechanisms for deglutathionylation are functionally equivalent: implications for redox systems biology
Source: Biosci Rep. 2015 Feb 25;35(1):e00173. doi: 10.1042/BSR20140157 (PMC4340274; doi:10.1042/BSR20140157)
Supplement: Supplementary data [file bsr035e173ntsadd.pdf]

The glutaredoxin mono- and di- thiol mechanisms for deglutathionylation are functionally equivalent: implications for redox systems biology.

Supplementary Information

Lefentse N. Mashamaite, Johann M. Rohwer[a] and Ché S. Pillay

School of Life Sciences, University of KwaZulu-Natal, South Africa, Carbis Road, Pietermaritzburg, 3201, South Africa.

[a] Department of Biochemistry, Stellenbosch University, Private Bag X1, Matieland, 7602 Stellenbosch, South Africa.

Shown below are the kinetic parameters used to build the computational models of the glutaredoxin system described in the accompanying paper. The full details of the kinetic model of the *E. coli* glutaredoxin system may be found in [1]. The kinetic models of the yeast glutaredoxin system were based on data presented by Li *et al.* [2] and the parameters and concentrations used in these models are shown in Table S1. PySCeS and SBML versions of the models are also included in the Supplementary Information.

Table S1 Kinetic parameters and species concentrations used for the yeast Grx 1 and Grx 2 models of Li *et al.* [2].

	Value		Reference
	**Grx1**	**Grx2**	
**Metabolite**			
NADPH	250 µM	250 µM	[2]
NADP	1 µM	1 µM	[2]
GSH	998 µM	998 µM	[2]
GSSG	1 µM	1 µM	[2]
HED	70 µM	70 µM	[2]
**Redoxin**			
$Grx(SH)_2$	0.12 µM	0.02 µM	[2]
Grx(SS)	0.12 µM	0.02 µM	[2]
**Glutathione reductase**			
K_{NADPH}	15 µM	15 µM	[3]
K_{GSSG}	74.6 µM	74.6 µM	[3]
k_{cat}	900 s^{-1}	900 s^{-1}	[3]
[Glutathione reductase]	0.02 µM	0.02 µM	[2]

References

1. Pillay, C. S., Hofmeyr, J. H., Olivier, B. G., Snoep, J. L. & Rohwer, J. M. (2009) Enzymes or redox couples? The kinetics of thioredoxin and glutaredoxin reactions in a systems biology context, *Biochem J.* **417**, 269-75.
2. Li, W. F., Yu, J., Ma, X. X., Teng, Y. B., Luo, M., Tang, Y. J. & Zhou, C. Z. (2010) Structural basis for the different activities of yeast Grx1 and Grx2, *Biochimica et biophysica acta.* **1804**, 1542-7.
3. Yu, J. & Zhou, C. Z. (2007) Crystal structure of glutathione reductase Glr1 from the yeast Saccharomyces cerevisiae., *Proteins.* **68**, 972-979.
